# Measuring psychological flexibility to promote wellbeing: validation of the Spanish Psy-Flex in a healthy sample

**DOI:** 10.3389/fpsyg.2026.1808313

**Published:** 2026-04-29

**Authors:** Antonio Crego, Sara Yela-Gómez, José Ramón Yela, Francisco J. Ruiz, Andrew T. Gloster

**Affiliations:** 1 Faculty of Psychology, Pontifical University of Salamanca, Salamanca, Spain; 2 Fundación Universitaria Konrad Lorenz, Bogotá, Colombia; 3 Faculty of Behavioral Sciences and Psychology, University of Lucerne, Lucerne, Switzerland

**Keywords:** psychological flexibility, Psy-Flex, mental health, wellbeing, reliability, validity, network analysis

## Abstract

**Introduction:**

In a context in which psychological flexibility has been revealed to be a pivotal process in psychological well-being, the Psy-Flex scale has emerged as a theoretically based, context-sensitive, brief scale that can be used in different settings and has good psychometric properties.

**Methods:**

This study aims at analyzing the psychometric properties of the Spanish translation of the Psy-Flex, based on the responses of a sample of 170 healthy individuals. Standard psychometric methods and network analysis were used to evaluate the behavior of the adapted scale.

**Results:**

The analyses on the six items of the Spanish Psy-Flex yielded a single-factor structure (χ^2^ = 20.435, *df* = 8, *p* = 0.009; χ^2^*/df* = 2.554; *GFI* = 0.961; *NFI* = 0.952; *TLI* = 0.943; *CFI* = 0.970: *SRMR* = 0.046: *RMSEA* = 0.096, *RMSEA* 90% CI = 0.045, 0.148, *PCLOSE* = 0.065), good internal consistency (Cronbach’s *α* = 0.839; 95% CI = 0.812, 0.866), and high consistency across time points (ICC = 0.887; 95% CI = 0.852, 0.916). The scale scores were associated with a wide range of measures related to psychological flexibility and inflexibility, well-being and mental health. Network analysis further supported the idea that the Psy-Flex items form a coherent structure.

**Discussion:**

The Spanish version of the Psy-Flex is a psychometrically adequate scale for use in a population without mental health problems as a measure of psychological flexibility.

## Introduction

At its heart, psychological flexibility is about how well a person can navigate life’s twists and turns. It involves being aware of and anchored in the present, aligning one’s perspective with what the person deeply cares about, being open to one’s inner world and external environment, and effectively engaging in meaningful courses of action (Hayes, 2019). This adaptability allows individuals to focus on behaviors that lead to a sense of purpose and self-worth, even in the midst of harsh or adverse circumstances. In essence, psychological flexibility refers to fully engaging with the present moment and it involves adapting or maintaining behavior, based on the context requirements, to align with chosen values (Hayes et al., 2012).

Based on this fundamental idea and on results from clinical research, Hayes et al. (1999, 2012) have created the Hexaflex model outlining six key dimensions of psychological flexibility: creating distance from unhelpful private experiences (cognitive defusion), allowing space for potentially disturbing inner events (acceptance), focusing on the present moment (mindfulness), developing a perception of the self as the context where experiences unfold (self-as-context), clarifying one’s personal values (presence of meaning), and value-consistent behavior (committed action). Psychological inflexibility, by contrast, involves experiential avoidance, cognitive fusion, a failure to engage with the present moment, an overidentification with the conceptualized self, a lack of clarity regarding one’s values, and a failure to commit with action (Ruiz et al., 2024). These flexibility and inflexibility processes relate directly to fundamental psychological dimensions like emotion, thought, attention, the self, motivation, and outward behavior (Hayes, 2019; Hayes et al., 2020; Hofmann et al., 2021).

The construct of ‘psychological flexibility’ is central in Acceptance and Commitment Therapy (ACT) (Hayes et al., 1999, 2012), a contextual behavioral psychotherapy framework that has proven efficacy across a wide array of mental health conditions (Gloster et al., 2020). From its inception, ACT has regarded psychological flexibility and its maladaptive counterpart, psychological inflexibility, as transdiagnostic processes involved in psychological well-being and psychological suffering, respectively (Hayes et al., 1999, 2012).

Empirical research has highlighted the role of psychological flexibility as a mechanism of change involved in the effectiveness of ACT (Lin et al., 2018) and cognitive behavioral therapy (CBT) (Åkerblom et al., 2021). Furthermore, psychological flexibility has been revealed as a key element for one’s mental health and life satisfaction (Kashdan and Rottenberg, 2010; Lucas and Moore, 2020). On the other hand, current research has found consistent evidence for experiential avoidance, a component of inflexibility, as a process implicated in a broad range of psychological problems, beyond specific diagnoses (Akbari et al., 2022; Boulanger et al., 2010). Experiential avoidance refers to a person’s inclination to avoid potentially distressing internal states, such as thoughts, emotions, memories and bodily sensations. Despite the immediate comfort it may provide, such avoidance can have negative long-term effects, preventing someone from living according to their deeply held values and achieving their life goals (Hayes et al., 2012). In this regard, avoiding potentially disturbing inner experiences is connected with poorer mental health (Chawla and Ostafin, 2007), and adverse effects on wellbeing (Machell et al., 2015).

Despite these outstanding results, research has also pointed to important methodological limitations of psychological flexibility studies. While acceptance and experiential avoidance are often cited as examples of psychological flexibility and psychological inflexibility, respectively, these terms are frequently used interchangeably in research (Bond et al., 2011; Wolgast, 2014). However, as Cherry et al. (2021) have remarked, the common practice of inferring psychological flexibility from the absence of psychological inflexibility is questionable. In this regard, the Acceptance and Action Questionnaire (AAQ-II), developed by Bond et al. (2011), is an instrument commonly used to measure the construct of psychological flexibility but originally intended to measure psychological inflexibility, defined as a pattern in which behavior is rigidly controlled by thoughts, feelings, and other inner experiences, or by the desire to avoid them, to the detriment of more adaptive, valued and meaningful actions (Levin et al., 2014). However, the AAQ-II, and other commonly used instruments, have been criticized for incompletely capturing the psychological (in)flexibility construct, as their items may refer to ‘values’ in an abstract way but do not assess how people respond to distress in the context of pursuing a valued course of action (Doorley et al., 2020). Furthermore, the discriminant validity of the AAQ-II is a matter of debate. Doubts have been raised that the instrument is likely to be a measure of neuroticism and negative affect (Tyndall et al., 2019; Wolgast, 2014). In contrast, other researchers have found evidence supporting the discriminant validity of the AAQ-II as an instrument measuring only psychological inflexibility that should not be interpreted as measuring psychological flexibility or experiential avoidance (Ruiz et al., 2024).

In their review of 12 measures of psychological flexibility, Cherry et al. (2021) found that most of them had limitations that may limit the degree to which research on this construct can be viewed as reliable and valid. They defended that the Personalized Psychological Flexibility Index (PPFI) (Kashdan et al., 2020) was the best available psychological flexibility measure considering both its psychometric qualities and its conceptual grounding. However, this measure is based on a somewhat different theoretical framework from that of ACT and areas of improvement have been found at the item level (Jo et al., 2023a).

In this context, the PsyFlex scale developed by Gloster et al. (2021) attempts to provide researchers with an instrument that covers all facets of the psychological flexibility construct, as understood in the ACT approach, and is context-sensitive (i.e., not implicitly measuring flexibility as a trait or without reference to a limited time frame). The Psy-Flex is an assessment measure consisting of six items, each referring to one of the six core processes proposed in the ACT Hexaflex (Hayes et al., 2012). In addition, the items are formulated to assess the presence of each flexibility skill, rather than its absence. Originally, the scale presented a unifactorial structure and excellent reliability. Moreover, the scale presented solid evidence of convergent, divergent, and incremental validity (Gloster et al., 2021). Subsequent studies adapting the scale to other languages, such as Korean (Jo et al., 2023b), Chinese (Li et al., 2024; Fang et al., 2024), Portuguese (Cunha et al., 2024; Neto et al., 2024) and Hebrew (Gur et al., 2024) have confirmed the scale’s single-factor structure, high reliability, and generally good psychometric properties. As expected, the Psy-Flex is positively linked to flexibility-related variables such as mindfulness, feeling meaning in life, and values and negatively connected with inflexibility-related variables such as experiential avoidance and cognitive fusion (Fang et al., 2024; Gloster et al., 2021; Gur et al., 2024). In addition, the original Psy-Flex and the translated versions have presented correlations with well-being, life satisfaction and mental health-related outcomes. In sum, the Psy-Flex stands out as a brief measure, with good psychometric properties and useful in clinical and research contexts. This instrument, which has been already translated into 20 languages (Association for Contextual Behavioral Science, n.d.), is of great interest to the contextual behavioral science community.

### The present study

Currently, there are two Spanish translations of the Psy-Flex scale. The first one appeared in 2021; however, it has not yet undergone empirical analysis (Gloster et al., 2021; Spanish translation by Ruiz et al., 2021). The second version, developed by Navarrete et al. (2025), translated the scale items slightly differently, and its psychometric properties were analyzed in a sample of people experiencing chronic pain. In this context, our study is aimed at analyzing the psychometric properties of the Psy-Flex, as translated by Ruiz et al. (2021), in a sample of healthy individuals. Specifically, we examine its factor structure, internal consistency, and temporal stability (test–retest reliability). Additionally, we explore the scale’s construct validity by examining its divergent, discriminant and convergent validity. To further validate the instrument, we assess criterion validity through correlations with established measures of psychological (in)flexibility —distinguishing between flexibility (i.e., mindfulness, self-compassion, presence of meaning, and behavioral activation) and inflexibility (i.e., experiential avoidance and cognitive fusion)— and indicators of well-being and mental health (i.e., stress, anxiety, depression, positive and negative affect, happiness, and life satisfaction). Finally, a network analysis of the six items is conducted to explore the internal interplay and relative importance of the Hexaflex processes within the scale.

Based on previous literature, we proposed the following hypotheses:

*H1:* The Spanish Psy-Flex will demonstrate a single-factor structure with adequate fit.

*H2:* The scale will show good internal consistency and high temporal stability over a one-year period.

*H3:* Regarding construct validity, we anticipate that psychological flexibility scores will be independent of sex and age (divergent validity). Furthermore, we expect the Psy-Flex to demonstrate discriminant validity by explaining more variance in its items than it shares with other related constructs, and convergent validity by capturing an amount of variance greater than that due to measurement error.

*H4:* The Psy-Flex will show significant correlations with the examined variables: positive associations with well-being and psychological flexibility-related measures, and negative associations with psychological distress and psychological inflexibility-related variables.

*H5:* The network analysis will reveal that the six items form a single connected component, showing a cohesive structure of conditional dependencies that supports the conceptualization of psychological flexibility as an interdependent construct.

Establishing the reliability and validity of the Spanish Psy-Flex in a healthy population will allow us to deepen our knowledge about an instrument for measuring psychological flexibility with great potential for research.

## Methods

### Sample

A sample consisting of 170 subjects was analyzed, with a predominance of the female gender at 75.9%. The participants’ average age was 42.81 years, with a standard deviation of 15.51 and an age range from 18 to 65 years. Regarding educational level, the following distribution was observed: 1.8% reported having a basic education; 13.5% completed professional training or secondary education; 55.3% achieved undergraduate university studies; and 29.4% obtained postgraduate studies. Most of the participants were employed (61.2%), followed by students (15.9%), unemployed persons (14.7%), retirees (7.6%), and 0.6% who were classified into other categories, including those with functional disabilities.

### Procedure

This study uses data from a larger research project (MSC-Health, registered at ClinicalTrials.gov with ID: NCT05695586), whose methodological details have been previously published elsewhere (Crego et al., 2025). Specifically, it mainly utilizes scores from the pre-intervention measurement time of the MSC-Health project, which were collected in January–February 2023 through an online questionnaire survey. To evaluate the scale’s temporal stability, we also utilized data gathered across five assessment points: pre-intervention (T1), post-intervention (T2), and at 3-month (T3), 6-month (T4), and 12-month (T5) follow-ups. The present study includes participants who were assigned to the experimental conditions and the control group. The research aimed to study a healthy general population aged 18–65 years who had not suffered from or were suffering from a psychiatric disorder, were not undergoing psychiatric or psychological treatment, and did not have a severe medical disorder.

We recruited study participants in Salamanca, Spain, during November and December 2022. Recruitment occurred through local media advertisements and social media postings. In January 2023, prospective candidates attended two in-person group meetings where they received comprehensive information about the project, including details on ethical approval (CEI 07/22/2019 from Pontifical University of Salamanca, Spain), data handling, and privacy. All participants provided informed consent. Additionally, participants in the experimental groups received a 30€ compensation at each measurement point to encourage motivation and commitment to the broader study requirements, such as attendance at training sessions and biological assessments.

### Instruments

Psychological flexibility was assessed utilizing the Psy-Flex scale (Gloster et al., 2021; Spanish translation by Ruiz et al., 2021). This instrument comprises six items, each rated on a 5-point Likert-type scale, ranging from 1 (Never) to 5 (Very often), with higher scores indicating greater psychological flexibility. The internal consistency, as measured by Cronbach’s alpha, was 0.839 in the present study.

The Spanish version of the 26-item Self-Compassion Scale (SCS; Garcia-Campayo et al., 2014), based on Neff (2003) conceptualization, was employed to measure self-compassion. This construct encompasses self-kindness, recognition of shared human experience, and mindful awareness, as opposed to self-criticism, isolation, and over-identification with distressing internal states. Participants rated items on a 5-point Likert-type scale, from 1 (Never) to 5 (Always), with higher scores reflecting enhanced self-compassion. In the present study, Cronbach’s alpha was 0.921.

The Mindful Attention Awareness Scale (MAAS; Brown and Ryan, 2003; Spanish version by Soler et al., 2012) was used to measure general mindfulness capacity. This 15-item scale utilized a 6-point Likert-type response format, from 1 (Almost always) to 6 (Almost never), with higher scores denoting higher levels of mindfulness. In this study, internal consistency (Cronbach’s alpha) was 0.897.

The Presence of Meaning subscale (5 items) from the Meaning in Life Questionnaire (MLQ; Steger et al., 2006; Spanish translation, developed by Steger and Zaccagnini, available at the original author’s website: http://www.michaelfsteger.com/wp-content/uploads/2013/03/MLQ-Spanish.doc) was utilized to assess perceived meaning in life. Items were rated on a 7-point Likert-type scale, from 1 (Absolutely true) to 7 (Absolutely untrue), with higher scores representing higher levels of experienced meaning. In the present study, Cronbach’s Alpha was 0.865.

Cognitive fusion was assessed using the Cognitive Fusion Questionnaire (CFQ; Gillanders et al., 2014; Spanish version by Romero-Moreno et al., 2014), a 7-item scale with responses ranging from 1 (Never) to 7 (Always), with higher scores reflecting increased cognitive fusion. In the current study, Cronbach’s alpha was 0.920.

Psychological inflexibility was measured using the Acceptance and Action Questionnaire-II (AAQ-II; Bond et al., 2011; Spanish version by Ruiz et al., 2013), a 7-item scale assessing the unwillingness to engage with and accept challenging internal experiences. Items were rated on a 7-point Likert-type scale, from 1 (Never true) to 7 (Always true), with higher scores denoting higher levels of inflexibility. In the present study, Cronbach’s alpha was 0.876.

Behavioral activation was assessed using the Activation subscale (7 items) of the Behavioral Activation for Depression Scale (BADS; Kanter et al., 2007; Spanish version by Barraca et al., 2011), which measures goal-directed activation. Items were rated on a 7-point Likert-type scale, from 0 (Not at all) to 6 (Completely), with higher scores indicating greater behavioral activation. In the present study, Cronbach’s alpha was 0.860.

Depression and anxiety were measured using the Hospital Anxiety and Depression Scales (HADS; Zigmond and Snaith, 1983; Spanish version by Terol Cantero et al., 2007), a 14-item screening instrument with separate subscales for anxiety and depression (7 items each). Items were rated on a 4-point scale (0–3), with higher scores reflecting greater symptom severity. In the present study, Cronbach’s alpha was 0.831 for the anxiety subscale; for the depression subscale, Cronbach’s alpha was 0.793.

Perceived stress was measured using the Perceived Stress Scale (PSS; Cohen et al., 1983; Spanish version by Remor, 2006). This is a 14-item scale with responses ranging from 0 (never) to 4 (very often), with higher scores indicating greater perceived stress. In this study, Cronbach’s alpha was 0.892.

Positive and negative affect were measured using the Positive and Negative Affect Schedule (PANAS; Watson et al., 1988; Spanish version by López-Gómez et al., 2015), comprising 10 items for each affect dimension. Items were rated on a 5-point Likert-type scale, from 1 (Very slightly or not at all) to 5 (Extremely), with higher scores reflecting greater positive or negative affect. In the present study, Cronbach’s alpha was 0.906 for positive affect; for negative affect, Cronbach’s alpha was 0.898.

Subjective happiness was measured using the Subjective Happiness Scale (SHS; Lyubomirsky and Lepper, 1999; Spanish version by Extremera et al., 2014), a 4-item scale with 7-point Likert-type responses, ranging from 1 to 7, with higher scores representing higher levels of subjective happiness. In the current study, Cronbach’s alpha was 0.853.

Satisfaction with life was measured using the Satisfaction with Life Scale (SWLS; Diener et al., 1985; Spanish version by Vázquez et al., 2013), a 5-item scale with 7-point Likert-type responses ranging from 1 (Strongly disagree) to 7 (Strongly agree), with higher scores denoting having more life satisfaction. In the present study, Cronbach’s alpha was 0.887.

### Statistical analyses

Preliminary analyses included the calculation of item-level means and standard deviations and bivariate correlations among the six Psy-Flex items to examine their descriptive characteristics and initial associations. Additionally, it should be noted that there were no missing values in the dataset, as the online questionnaire survey utilized a forced-response format for all items.

First, Confirmatory Factor Analysis (CFA) was used to analyze the factor structure of the Psy-Flex scale (H1). This analysis was performed with AMOS 24 software for structural equation modeling (IBM, Armonk, USA). To evaluate the model fit, the following indices and cut-off values were considered (Browne and Cudeck, 1993; Hu and Bentler, 1999): χ^2^/*df* ≤ 3; Goodness of Fit Index (GFI), Normed Fit Index (NFI), Tucker-Lewis Index (TLI), and Comparative Fit Index (CFI) ≥ 0.90; Standardized Root Mean Square Residual (SRMR) ≤ 0.08; and Root Mean Square Error of Approximation (RMSEA) ≤ 0.10. To ensure the adequacy of the sample size (*N* = 170), a power analysis for the CFA was performed following the *RMSEA*-based method proposed by MacCallum et al. (1996), testing the null hypothesis of exact fit (*RMSEA* = 0) against the alternative hypothesis of poor fit (*RMSEA* ≥ 0.10).

Second, reliability analyses were carried out to evaluate the scale’s internal consistency and temporal stability (H2). Internal consistency was assessed using the total sample (*N* = 170), by calculating Cronbach’s alpha for the 6 items of the Psy-Flex. Values were interpreted as acceptable if *α* > 0.70 and good if α > 0.80 (Field, 2024). The confidence interval of the alpha statistic was calculated following the procedures described in Feldt et al. (1987). Item-total correlations (interpreted as adequate if *r* > 0.30) were used to evaluate the homogeneity of the Psy-Flex items, indicating the extent to which each item is associated with the rest of the scale (Field, 2024). In the same way, alpha coefficients if each item were deleted from the scale were assessed, in order to check whether any of the items produced a decrease in the internal consistency of the scale and therefore in its reliability. Temporal stability was calculated using the total scores of the Psy-Flex scale, which had been administered to the participants at 5 different times (pre-, post-, 3 months, 6 months, and 1-year). For this purpose, the intraclass correlation coefficient (ICC; two-way random effects model; type of measure: consistency) was used and correlations between Psy-Flex total scores at the 5 measurement points were calculated. Furthermore, to account for the structural properties of the model, Composite Reliability (CR) was calculated based on the standardized factor loadings from the CFA, using as a threshold the recommended value of 0.70 (Hair et al., 2019).

Third, construct validity was analyzed (H3). Divergent validity was examined to ensure the construct’s independence from sociodemographic variables. Independent samples *t*-tests were used to compare Psy-Flex scores by sex, and Pearson’s correlation was used to evaluate the association with age. These analyses were conducted using SPSS 22 (IBM, Armonk, USA). Convergent validity was assessed through the Average Variance Extracted (AVE), applying a recommended threshold of 0.50; however, following Fornell and Larcker (1981), values slightly below this cut-off were considered acceptable provided that the CR exceeded 0.60. Finally, discriminant validity was examined using the Fornell and Larcker (1981) criterion, which requires the square root of the AVE for the construct to be greater than its correlations with any other variable in the study.

Fourth, the criterion validity of the scale was assessed by analyzing the relationships of the Psy-Flex with other well-established scales related to psychological (in)flexibility and well-being (H4). These scales referred to measures associated with the six Hexaflex components, such as flexibility-related processes (i.e., mindfulness, presence of meaning, behavioral activation, self-compassion) and inflexibility-related variables (i.e., cognitive fusion and experiential avoidance), and to well-being and mental health-related variables (i.e., subjective happiness, life satisfaction, positive and negative affect, perceived stress, anxiety, and depression). Pearson’s correlations between the Psy-Flex total scores and the aforementioned variables were used to assess the validity of the scale, and their magnitude was interpreted according to Cohen’s (1988) criteria: 0.10 (small), 0.30 (medium), and 0.50 (large). In addition, the correlations between each of the six Psy-Flex items and the variables referring to psychological (in)flexibility-related processes and outcome variables (i.e., well-being and mental health) were analyzed. All the abovementioned analyses were conducted using SPSS 22 (IBM, Armonk, USA).

Finally, an item-level network analysis was conducted to further explore the internal structure and relative importance of the Psy-Flex items. The network was estimated using the bootnet and qgraph packages in R. Specifically, a regularized partial correlation network was computed using the Graphical Least Absolute Shrinkage and Selection Operator (glasso) in combination with the Extended Bayesian Information Criterion (EBIC) with a tuning parameter of *γ* = 0.5. This approach ensures a parsimonious model; the LASSO algorithm applies a regularization penalty that shrinks redundant associations to exactly zero, identifying only unique conditional dependencies. In the resulting visualization, produced using the ‘circle’ layout to facilitate the comparison of associations, items were represented as nodes and their conditional dependencies as edges. Following current recommendations (Bringmann et al., 2019), centrality was assessed through the standardized node strength, as it is considered the most stable and reliable index for psychological networks. To ensure statistical robustness, the model was assessed via two bootstrapping procedures (1,000 iterations; fixed random seed): (a) a non-parametric bootstrap to estimate 95% Confidence Intervals (CIs) for edge weight accuracy, and (b) a case-dropping bootstrap to calculate the Centrality Stability (CS) coefficient (Epskamp et al., 2018). According to established standards, a CS-coefficient above 0.25 is considered acceptable, while values above 0.50 are considered ideal.

## Results

### Descriptive and bivariate correlations between Psy-Flex items

Considering that the Psy-Flex scale scores range from 1 to 5, it can be said that the participants generally reported moderate levels of psychological flexibility, with mean scores close to the midpoint value of 3.

As shown in Table 1, the Psy-Flex scale items were significantly associated with each other and with the total scale scores. The item referring to ‘commitment to action’ presents the lowest correlations with the rest, except in the case of the item referring to ‘values’.

**Table 1:** Descriptive statistics and bivariate correlations among Psy-Flex items.

Psy-Flex items	*Mean*	*Sd*	1	2	3	4	5	6
1. Item 1 (Mindfulness)	3.40	1.242						
2. Item 2 (Acceptance)	2.94	1.094	0.411^**^					
3. Item 3 (Defusion)	2.64	1.123	0.473^**^	0.650^**^				
4. Item 4 (Self)	2.60	1.101	0.477^**^	0.553^**^	0.759^**^			
5. Item 5 (Values)	3.21	1.125	0.449^**^	0.434^**^	0.518^**^	0.588^**^		
6. Item 6 (Committed action)	4.06	0.861	0.343^**^	0.274^**^	0.248^**^	0.250^**^	0.470^**^	
7. Psy-Flex total scores	3.14	0.816	0.725**	0.749**	0.828**	0.823**	0.774**	0.545**

*N* = 170; **Correlation is significant at *p* < 0.01; ***Correlation is significant at *p* < 0.001.

### Factor structure of the Psy-Flex scale (confirmatory factor analysis, CFA)

**Figure 1:**
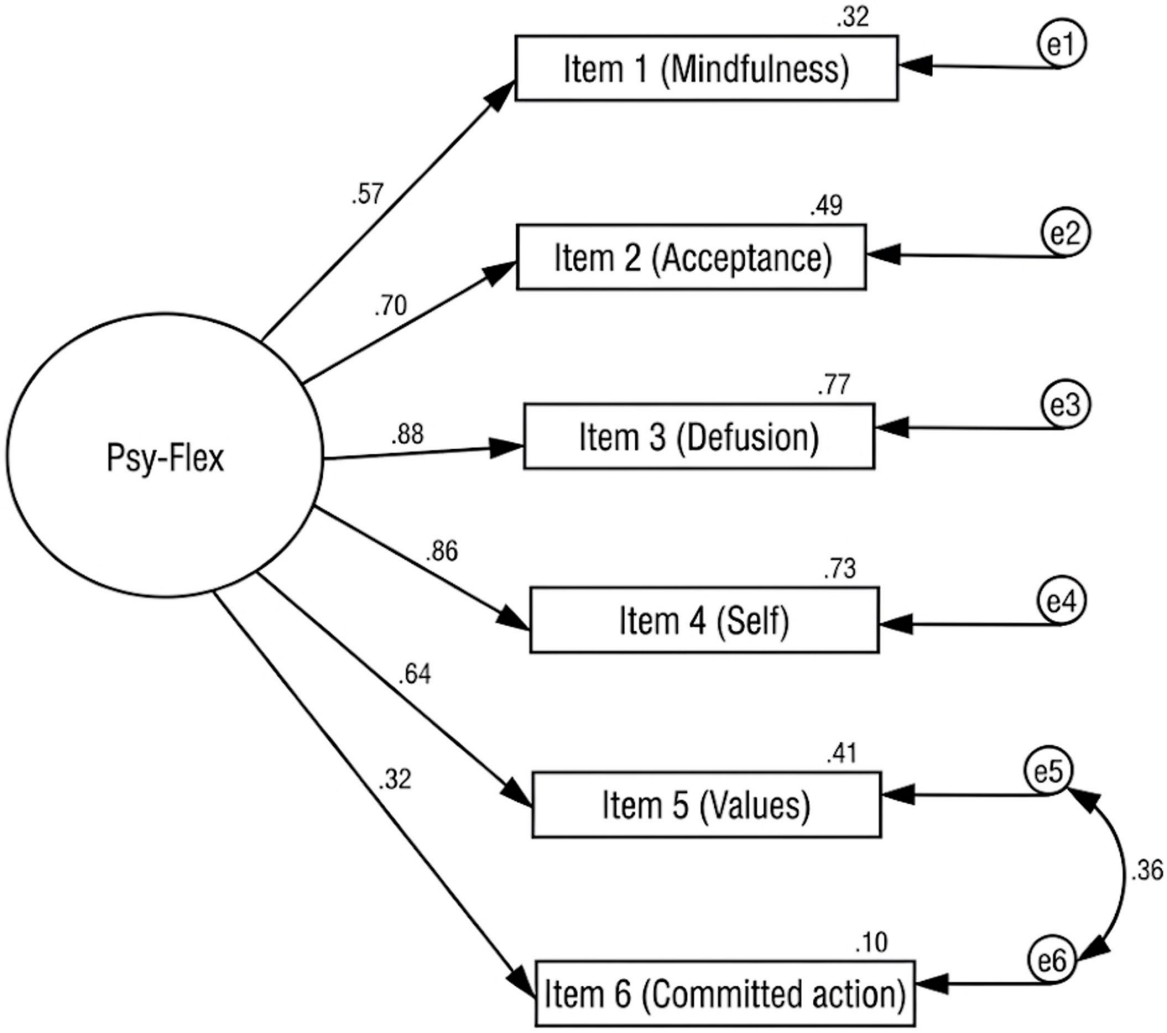
One-factor CFA model for the Psy-Flex. Standardized regression weights are shown on the paths from the latent construct to the observed items. Values above the items represent estimated explained variances. The bidirectional arrow between error terms e5 and e6 indicates the modeled covariance between the residuals of the ‘values’ and ‘committed action’ items.

A one-factor structure of Psy-Flex was tested using CFA. As suggested by the modification indexes and the results from previous research (Gloster et al., 2021; Navarrete et al., 2025), the error correlation between the items referring to ‘values’ and ‘commitment to action’ was allowed. The unifactorial solution presented a reasonable fit to the data (χ^2^ = 20.435, *df* = 8, *p* = 0.009; χ^2^*/df* = 2.554; *GFI* = 0.961; *NFI* = 0.952; *TLI* = 0.943; *CFI* = 0.970: *SRMR* = 0.046: *RMSEA* = 0.096, *RMSEA* 90% CI = 0.045, 0.148, *PCLOSE* = 0.065). Item loadings on the latent factor are presented in Figure 1. The single-factor model with *df* = 8 and a sample of *N* = 170 yielded a post-hoc power of 0.75 to detect an exact fit.

### Reliability analysis

The reliability analyses (internal consistency) carried out on the total sample (*N* = 170) showed that the single-factor scale presented a Cronbach’s *α* = 0.839 (95% CI = 0.812, 0.866). If any of the items of the scale were eliminated, the alpha statistic would always present values lower than that obtained of 0.839, except in the case of eliminating the item referring to ‘commitment to action’, where it would improve slightly to α = 0.847. The corrected item-total correlations were *r* = 0.564 (item ‘mindfulness’), *r* = 0.622 (item ‘acceptance’), *r* = 0.730 (item ‘defusion’), *r* = 0.724 (item ‘self’), *r* = 0.652 (item ‘values’) and *r* = 0.403 (item ‘commitment to action’), being in all cases above the *r* value < 0.30 suggested by Field (2024) for the elimination of an item.

With respect to reliability understood as the stability of scale scores across different time points, the intraclass correlation coefficient (ICC; two-way random effects model; type of measure: consistency) was indicative of high consistency across time points, with ICC = 0.887 (95% CI = 0.852, 0.916). Correlations among Psy-Flex total scores across the five time points are shown in the Table 2. Since the longitudinal measures of psychological flexibility were obtained in the context of a broader research project where our participants received mindfulness-based interventions (*n* = 87) or were assigned to a control group (*n* = 32), we additionally calculated the ICC for each condition. As reported elsewhere (Crego et al., 2025), the Psy-Flex scores of participants who received mindfulness-based trainings significantly increased their psychological flexibility scores after 8 weeks and remained stable at that level during one year of mindfulness practice. In contrast, the flexibility scores in the control group remained stable across times of measure. Consistently, in the experimental conditions the Psy-Flex yielded an overall ICC = 0.880 (95% CI = 0.836, 0.916), with ICC = 0.666 (95% CI = 0.510, 0.772) for the 8-week training period when Psy-Flex scores increased and ICC = 0.902 (95% CI = 0.864, 0.932) during subsequent time periods when gains in psychological flexibility remained stable. In the experimental group, ICC = 0.916 (95% CI = 0.859, 0.955) was obtained across moments of measure over the one-year period.

**Table 2:** Test–retest reliability.

Time	1	2	3	4
Pre-intervention (T1)				
Post-intervention (T2)	0.519			
3-months follow-up (T3)	0.516	0.755		
6-months follow-up (T4)	0.560	0.669	0.731	
12-months follow-up (T5)	0.430	0.616	0.672	0.760

Pearson’s correlations among Psy-Flex total scores across five moments of measurement. *N* (listwise) = 119; All correlations are significant at *p* < 0.001.

Finally, the Psy-Flex demonstrated excellent structural reliability with a CR = 0.83, significantly exceeding the widely accepted threshold of 0.70. This indicates that the latent construct is robustly represented by its six items and that the measurement error is within acceptable limits.

### Construct validity

#### Divergent validity

A *t*-test for independent samples revealed that there are no significant differences between men (*Mean* = 3.33, *Sd* = 0.81) and women (*Mean* = 3.08, *Sd* = 0.81) in Psy-Flex scores (*t* = −1.732, *gl* = 169, *p* = 0.085). The age of the participants was also not found to be significantly associated with their Psy-Flex scores (*r* = −0.014, *p* = 0.853).

#### Convergent and discriminant validity

The one-factor model yielded an AVE of 0.47. Concerning discriminant validity, in our study the square root of the AVE for the Psy-Flex was 0.685, which exceeded all correlations between the Psy-Flex and the other studied constructs (Table 3).

**Table 3:** Pearson’s correlations of the Psy-Flex with psychological flexibility-related and outcome variables.

Variables	Psy-Flex Items						Psy-Flex total scores
	Item 1 (Mindfulness)	Item 2 (Acceptance)	Item 3 (Defusion)	Item 4 (Self)	Item 5 (Values)	Item 6 (Committed action)	
Psychological flexibility-related variables							
MAAS	0.403**	0.218**	0.386**	0.310**	0.338**	0.223**	0.426**
AAQ-II	−0.371**	−0.369**	−0.609**	−0.563**	−0.503**	−0.330**	−0.617**
CFQ	−0.334**	−0.339**	−0.593**	−0.575**	−0.441**	−0.225**	−0.567**
SCS	0.329**	0.387**	0.613**	0.591**	0.445**	0.289**	0.596**
MLQ-P	0.237**	0.184*	0.356**	0.350**	0.374**	0.319**	0.404**
BADS-A	0.324**	0.104	0.247**	0.349**	0.292**	0.309**	0.362**
Outcome variables							
PSS	−0.418**	−0.358**	−0.574**	−0.596**	−0.503**	−0.328**	−0.625**
HAD_ANXIETY	−0.385**	−0.395**	−0.545**	−0.535**	−0.436**	−0.260**	−0.577**
HAD_DEPRESSION	−0.259**	−0.291**	−0.476**	−0.366**	−0.378**	−0.262**	−0.455**
PA	0.328**	0.339**	0.488**	0.424**	0.431**	0.391**	0.534**
NA	−0.378**	−0.360**	−0.554**	−0.567**	−0.511**	−0.260**	−0.594**
SHS	0.289**	0.250**	0.464**	0.427**	0.463**	0.316**	0.494**
SWLS	0.237**	0.201**	0.300**	0.382**	0.414**	0.247**	0.398**

*N* = 170; *Correlation is significant at the 0.05 level (two-tailed); **Correlation is significant at the 0.01 level (2-tailed). MAAS: Mindful Attention Awareness Scale. AAQ-II: Acceptance and Action Questionnaire. CFQ: Cognitive Fusion Questionnaire. SCS: Self-compassion scale. MLQ-P: Meaning in Life Questionnaire (Presence of Meaning). BADS-A: Behavioral Activation for Depression Scale (Activation). PSS: Perceived Stress Scale. HAD: Hospital Anxiety and Depression Scales. PA: Positive affect. NA: Negative affect. SHS: Subjective Happiness Scale. SWLS: Satisfaction with life scale.

### Validity of criteria

The six items of the Psy-Flex were shown to be significantly associated with (in)flexibility process-related and outcome variables that were used to estimate the criterion validity of the scale. In relation to the process measures, six of them were selected which are common instruments to evaluate variables connected with the processes of the Hexaflex model (Hayes et al., 2012) and therefore may be in correspondence with the six Psy-Flex items. As can be seen in Table 3, in all cases, except for the correlation between the Psy-Flex item ‘acceptance’ and the behavioral activation measure (BADS-A), significant associations between the Psy-Flex items and the process variables considered were obtained.

As expected in our Hypothesis 4, the total scores of the Psy-Flex yielded significant correlations of medium to large size with each of the six process variables analyzed. Of all the scales that were examined, the AAQ-II demonstrated the strongest negative correlation with the Psy-Flex total scores (Table 3). It is also remarkable that consistent associations have been found between each Psy-Flex item and the scales measuring either the same psychological flexibility process or their corresponding inflexibility counterparts. Thus, Psy-Flex item 1 (‘mindfulness’) shows its highest positive correlation with the MAAS scale intended to evaluate mindfulness. The AAQ-II scale correlated strongly and negatively with the Psy-Flex items referring to ‘defusion’, ‘self’ and ‘values’, followed by the item on ‘acceptance’. The cognitive fusion scale CFQ presented its highest negative correlation with Psy-Flex item 3 (‘defusion’). The SCS scale, which measures self-compassionate attitudes, presents large positive correlations with Psy-flex items 4 (‘self’) and 3 (‘defusion’). The MLQ-Presence of Meaning scale presents its highest positive correlation with item 5 (‘values’). Finally, the BADS-A scale —referred to behavioral activation— was positively correlated, among its highest associations, with the Psy-Flex item referred to ‘commitment to action’, together with the items on ‘mindfulness’ and ‘self’.

The Psy-Flex scale (total scores), as well as separately each of the six items included in it, proved to be significant predictors of all the outcome variables considered (i.e., they were negatively associated with perceived stress, anxiety, depression, and negative affect; and positively connected with positive affect, happiness, and life satisfaction), with correlations ranging from medium (*r* ≈ 0.30) to large (*r* ≈ 0.50) size in most cases (Table 3).

### Network analysis of Psy-Flex items

Standardized strength values (*z*-scores) revealed that ‘defusion’ emerged as the most central node in the network (Strength = 1.25), indicating that it exhibits the strongest conditional associations with the rest of the psychological flexibility components. Other highly central nodes included ‘self as context’ (Strength = 0.92) and ‘values’ (Strength = 0.36). In contrast, ‘acceptance’ (Strength = −0.50) and ‘mindfulness’ (Strength = −0.75) showed below-average centrality, while the ‘committed action’ item exhibited the lowest node strength (Strength = −1.28), suggesting a more peripheral role within the item-level network structure.

**Figure 2:**
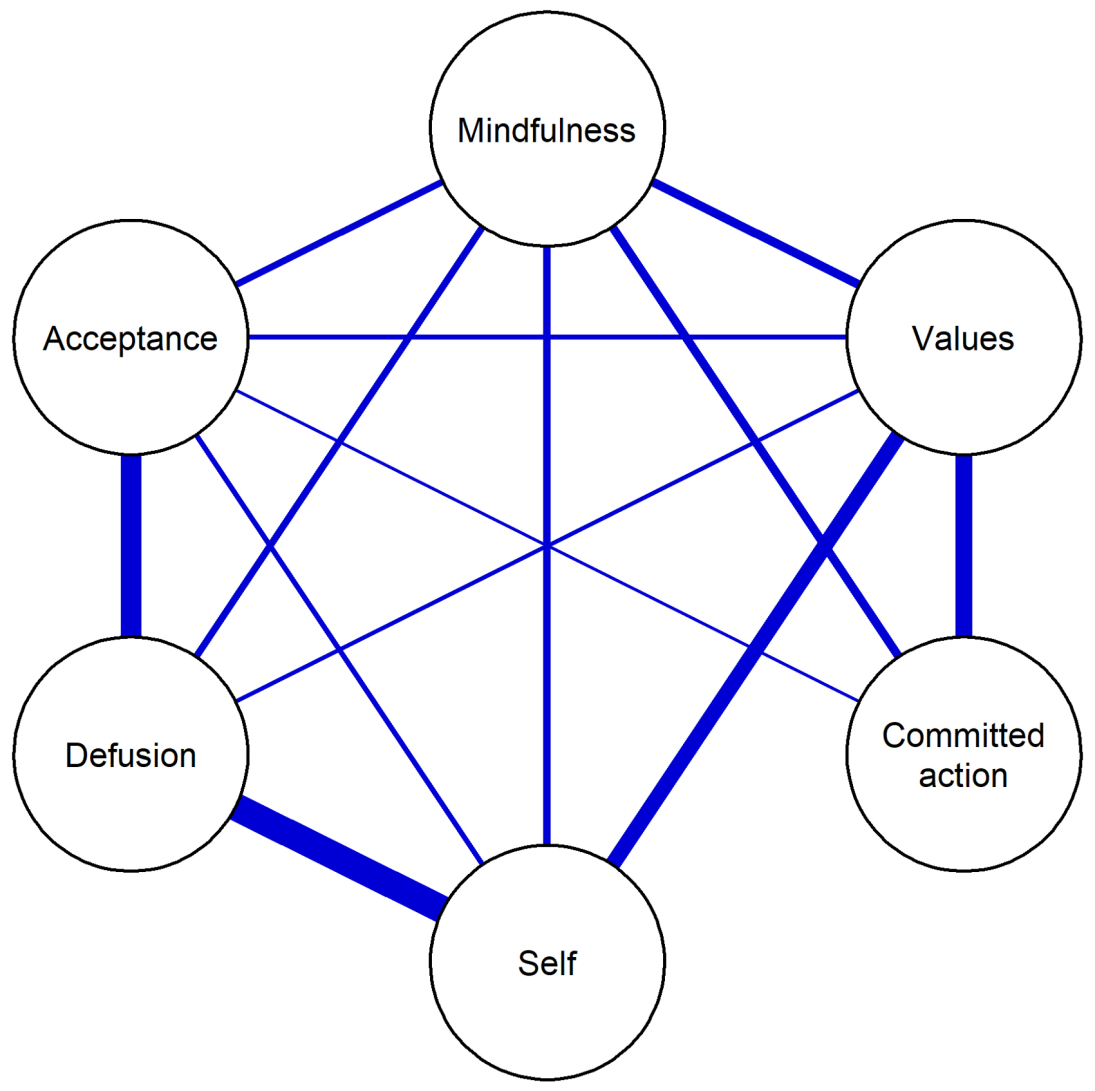
Regularized partial correlation network of the Psy-Flex components. Nodes represent the six components of psychological flexibility from the Psy-Flex instrument. Edges represent regularized partial correlation weights. Thicker lines indicate stronger conditional dependencies. Network estimated using the qgraph and bootnet packages in R with LASSO regularization and Extended Bayesian Information Criterion (EBIC, tuning parameter *γ* = 0.5). All edges are blue, representing positive unique conditional associations after regularization.

The specific edge weights (regularized partial correlations) for the associations between the items are detailed in Table 4 and represented in Figure 2. The strongest connections emerged between self and defusion (weight = 0.52), and between acceptance and defusion (weight = 0.38), while negligible conditional associations were successfully regularized to zero.

**Table 4:** Adjacency matrix of regularized partial correlations (edge weights) for the estimated 6-node Psy-Flex network.

Psy-Flex items	**1**	**2**	**3**	**4**	**5**
1. Item 1 (Mindfulness)					
2. Item 2 (Acceptance)	0.10				
3. Item 3 (Defusion)	0.11	0.38			
4. Item 4 (Self as context)	0.12	0.08	0.52		
5. Item 5 (Values)	0.14	0.07	0.06	0.28	
6. Item 6 (Committed action)	0.14	0.04	0.00	0.00	0.31

Values represent regularized partial correlations (edge weights) estimated using the EBICglasso algorithm with a tuning parameter of γ = 0.5. Values of 0.00 indicate that the association was shrunk to zero by the LASSO regularization, suggesting that the relationship between those specific nodes is redundant when accounting for the rest of the network structure.

**Figure 3:**
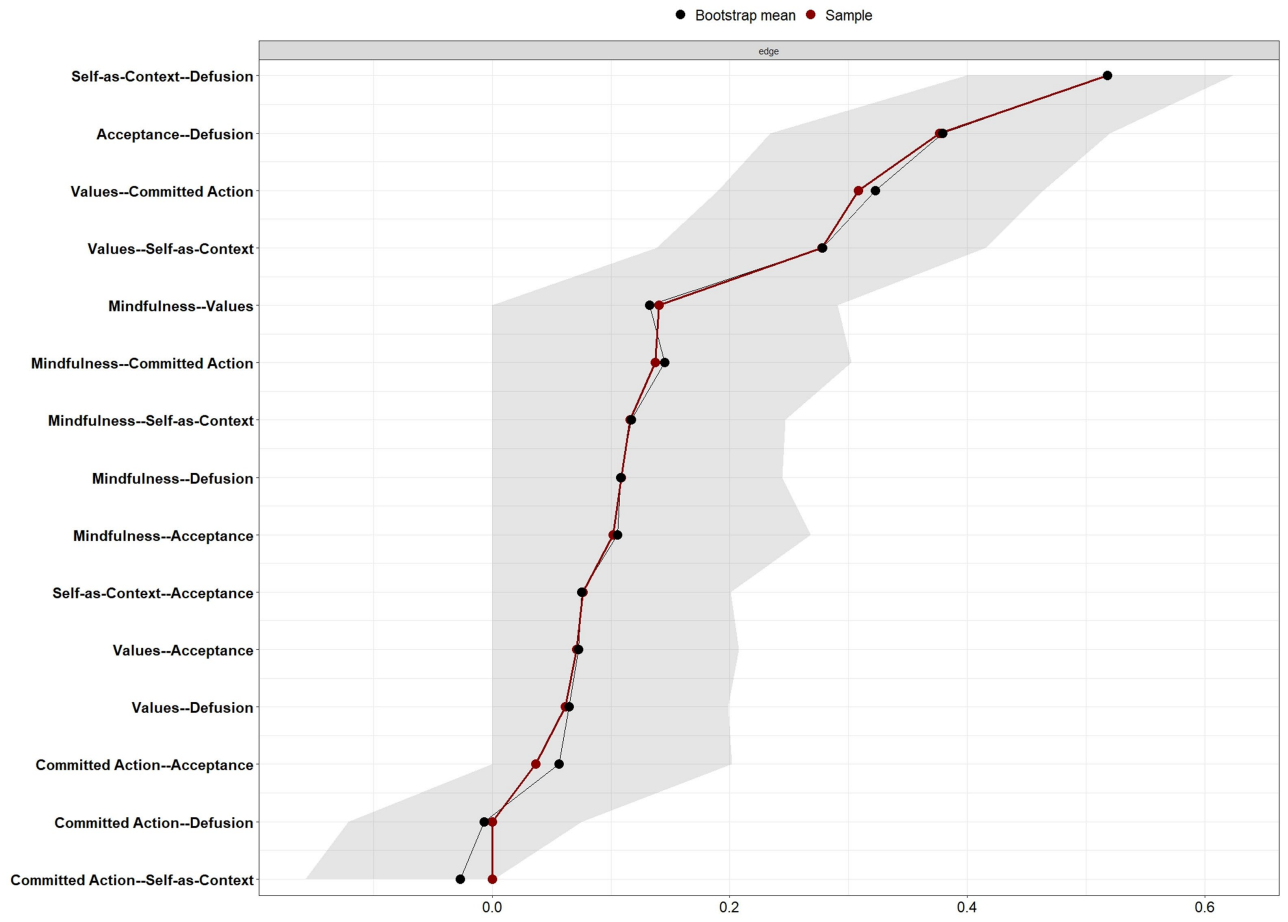
Accuracy of edge weight estimations for the Psy-Flex network. The plot displays the bootstrapped confidence intervals (CIs) for the estimated regularized partial correlations. The *y*-axis represents all potential edges in the network, ordered by their magnitude in the sample. The red line indicates the edge weights obtained from the original sample, while the black line represents the mean edge weights derived from 1,000 non-parametric bootstrap iterations. The gray shaded area represents the 95% non-parametric bootstrap CI.

The edge weight accuracy plot (Figure 3) displays the 95% confidence intervals (CIs) for all potential connections. The results indicate that the strongest edges in the sample (most notably ‘self’—‘defusion’ and ‘acceptance’—‘defusion’), remained robust, as their CIs did not overlap with zero. Furthermore, the close alignment between the sample values (red dots) and the bootstrap mean values (black dots) across all edges suggests that the network parameters are stable and were not significantly influenced by outliers. While some CIs for weaker edges showed greater overlap with zero, the overall hierarchy of the most central associations remained consistent, providing evidence for a reliable network structure within the current sample.

The case-dropping bootstrap analysis demonstrated excellent stability for the estimated network. The CS-coefficients were 0.67 for edge weights and 0.52 for node strength. Since both values surpass the recommended ideal threshold of 0.50, the results confirm that the network structure and centrality indices are highly robust, indicating that the current sample size is adequate.

## Discussion

The aim of this study was twofold. First, to evaluate the psychometric properties of the Spanish translation of the Psy-Flex (Ruiz et al., 2021); and second, to deepen our knowledge of the relationships between the Psy-Flex and other instruments commonly used to measure aspects of psychological (in)flexibility, well-being and mental health. Regarding the properties of the Psy-Flex, we found that the one-factor structure adequately fits the data. This is consistent with our Hypothesis 1 and with previous results obtained both in the original development of the scale (Gloster et al., 2021) and in successive language adaptations (Cunha et al., 2024; Fang et al., 2024; Gur et al., 2024; Jo et al., 2023b; Li et al., 2024; Navarrete et al., 2025). In our case, to achieve an optimal fit of the items to the one-factor structure, we allowed for correlation between the errors of item 5 (‘values’) and item 6 (‘committed action’). This strategy was employed by Gloster et al. (2021) when validating the original scale and by Navarrete et al. (2025) when studying the Spanish Psy-Flex with people experiencing chronic pain. Similarly, the Korean adaptation study of the Psy-Flex tested single-factor models with and without correlation between items 5 and 6. Initially, the one-factor model exhibited poor fit when error terms were uncorrelated. However, incorporating correlated residuals in the revised model led to a significant improvement in fit (Jo et al., 2023b). In their analysis of the properties of the Portuguese Psy-Flex, Cunha et al. (2024) also opted for a single factor structure model with the specification of a correlation between items 5 and 6 error terms. Theoretically, this association is justified by the functional interdependence of these two processes; as Gloster et al. (2021) argued, ‘committed action’ is inherently predicated upon the clarification of ‘values’, as purposeful behavior requires a predefined valued direction. Furthermore, a linguistic nuance likely contributes to this shared variance, as both items specifically employ the descriptor ‘important’ to assess their respective domains, which also occurs in the Spanish translation (Ruiz et al., 2021). This dual overlap—both conceptual and methodological—accounts for the specific covariance not captured by the primary latent factor, yet it reinforces the functional unity of the scale by acknowledging the essential bridge from internal valuation to behavioral enactment.

Regarding the reliability of the Spanish Psy-Flex, as expected in the Hypothesis 2, our results have yielded a Cronbach’s alpha (*α* = 0.834) indicating a high internal consistency of the items that constitute the scale. This finding is consistent with the results obtained in other studies such as the Chinese (*α* = 0.82) (Fang et al., 2024), Portuguese (*α* = 0.82) (Cunha et al., 2024), Hebrew (*α* = 0.91) (Gur et al., 2024), or Korean (*α* = 0.727) (Jo et al., 2023b).

An interesting contribution of our study is the analysis of the consistency of the Psy-Flex over a whole year, drawing on data from a larger research project in which five measurements were taken. In this sense, the overall ICC = 0.887 is indicative of a high consistency of responses over time, and is a result similar to that obtained for the Chinese version of the Psy-Flex (ICC = 0.843) over a one-month period, but higher than that obtained in the Portuguese case over a 4-week period (ICC = 0.62) (Cunha et al., 2024). This result could be pointing to the fact that —beyond the Psy-Flex being a context-sensitive measure— the maintenance of greater or lesser psychological flexibility levels could also be a response style with a certain temporal stability. In fact, after analyzing longitudinal data obtained in the context of a larger research project in which some participants received mindfulness-based trainings that increased their levels of psychological flexibility, we found that Psy-Flex is both sensitive to change following an intervention and stable when the intervention does not produce further gains or when there is no intervention.

Regarding Hypothesis 3, our results confirmed that the Psy-Flex scores are independent of sociodemographic variables such as sex and age. The absence of significant differences between men and women and the lack of a correlation with age provide strong evidence for the divergent validity of the scale. This is consistent with the original validation study by Gloster et al. (2021) and may suggest the usability of the instrument within the Spanish general population, indicating that it measures the individuals’ psychological flexibility regardless of their gender or stage of life. Furthermore, according to the Fornell and Larcker (1981) criteria, our results confirmed that the Psy-Flex explains more variance in its own items than it shares with other psychological constructs, providing evidence of its empirical distinctiveness (discriminant validity) as required for construct validation. With respect to convergent validity, although we obtained an AVE value slightly below the ideal 0.50 criterion, convergent validity may be considered adequate following the recommendations of Fornell and Larcker (1981). According to these authors, if the CR is higher than 0.60 —as in the present study—, a construct’s convergent validity is still statistically acceptable even if the AVE is less than 0.50.

As expected in Hypothesis 4, the Spanish Psy-Flex yielded positive correlations with other instruments measuring psychological flexibility processes and negative associations with their corresponding inflexibility-related counterparts. In this sense, it is noteworthy that Psy-Flex total scores have their strongest correlation —negatively in this case— with the AAQ-II, which is the most commonly used measure of psychological inflexibility. While the Chinese (Fang et al., 2024) and Korean (Jo et al., 2023b) adaptations of the scale found medium-sized correlations between the Psy-Flex and the AAQ-II, our results more closely resemble those of the original validation by Gloster et al. (2021) or the cross-cultural (Brazil-Portugal) validation (Neto et al., 2024) where a large-sized negative correlation was obtained. Also consistent with other validation studies (Gloster et al., 2021; Neto et al., 2024), we found strong negative correlations between the Spanish Psy-Flex and the CFQ, a measure of cognitive fusion. Interestingly, our study has found a strong association between the Psy-Flex and the SCS self-compassion scale. This relationship opens up promising avenues for research and joins the voices that are increasingly linking the two constructs (Weinstein, 2025; Yela and Crego, 2025).

In addition, as expected, we found medium-sized positive associations with other aspects of psychological flexibility, such as mindfulness, feeling meaning in life, and behavior activation.

The Psy-Flex is a tool that may be of great value in predicting psychological well-being and mental health, as indicated by its strong associations with variables such as perceived stress, anxiety, and positive and negative affect, and its medium-strong correlations with depression, happiness, and life satisfaction. Our results, once again, provide additional evidence and are consistent with those found in previous studies (Cunha et al., 2024; Fang et al., 2024; Gloster et al., 2021; Jo et al., 2023b; Neto et al., 2024).

Regarding Hypothesis 5, the network analysis revealed a cohesive structure where the six items were integrated into a single functional component. While the model is not fully saturated—as some associations were regularized to zero (e.g., between ‘acceptance’ and ‘committed action’)—this indicates conditional independence between certain processes once the influence of the rest of the network is accounted for. This may suggest that the coordination between more peripheral items is channeled through highly central nodes, such as ‘defusion’ and ‘self-as-context’. Rather than a simple ‘all-to-all’ interaction, the Spanish Psy-Flex appears to function through a specific architecture where core processes may account for the shared variance between the other components of the model. The peripheral position and lower node strength of ‘committed action’ deserve particular attention. Within the Hexaflex model, committed action represents the behavioral manifestation of the psychological flexibility repertoire. Unlike more internal processes—such as ‘defusion’ or ‘acceptance’—behavioral enactment is often contingent upon environmental opportunities and external barriers. Therefore, its relative marginality in the network may reflect that, in the current sample, acting in accordance with one’s goals could be a complex output influenced by situational factors beyond the purely psychological domain. Nevertheless, the significant error covariance between ‘values’ and ‘committed action’ found in the CFA, coupled with their direct edge in the network, may suggest that these processes function as a dyadic behavioral unit, where values provide the necessary direction for action to be considered a component of flexibility rather than mere activity.

Finally, it is also worth reflecting on the Psy-Flex —beyond its good psychometric properties— from a conceptual point of view. Cherry et al. (2021) propose that a consensus definition of psychological flexibility requires three core elements: managing interference or distress, acting to address that discomfort, and behaving in a way that aligns with situational demands and helps achieve personal goals or values. From our point of view, a careful reading of the Psy-Flex items shows that it is an instrument that meets these three requirements for an adequate definition of the construct. Moreover, the Psy-Flex is a measure built on the multicomponent definition of psychological flexibility proposed by the creators of ACT (Hayes et al., 2012; Hayes, 2019). In this regard, the fact that each of the items that constitute the scale refers to one of the processes identified in the Hexaflex model (Hayes et al., 2012) is a guarantee of the construct validity of the Psy-Flex.

### Practical implications

As is well known, the Psy-Flex is a tool with applications in research and clinical settings, whose usefulness is further enhanced by the fact that it is a brief instrument that requires only a few minutes to be completed by respondents. In addition, its good psychometric and conceptual properties make the Psy-Flex a tool with enormous potential and its use is expected to expand in the coming years.

Moreover, the identification of core variables in the network of process-related variables such as psychological flexibility (Psy-Flex), psychological inflexibility (AAQ-II) and self-compassion (SCS) has significant implications for the evaluation of individuals and the design of psychological interventions (Ciarrochi et al., 2024; Gloster et al., 2023; Kirby, 2025; Rutschmann et al., 2024). Focusing efforts on improving psychological flexibility and self-compassion could generate positive changes that extend to other areas of psychological functioning, given their central role in the network. The idea of a psychology focused on identifying mechanisms of change is in line with the recent postulates of Hayes et al. (2020) and Hofmann et al. (2021) and, in this regard, the availability of appropriate tools aimed at measuring psychological flexibility and inflexibility processes is of great importance.

### Limitations

Despite its contributions, our research is not without limitations. First, the sample used to carry out the validation study of the Spanish Psy-Flex, including 170 participants, is relatively small compared to sample sizes used in other scale adaptation studies. Even so, our study shows that the internal structure and psychometric properties of the Psy-Flex remain robust even in a smaller sample. Power analysis for the model (*df = 8*) yielded a value of 0.75, which is slightly below the conventional 0.80 threshold. However, as suggested by Wolf et al. (2013), the presence of several high-loading items (>0.70) provides substantial ‘anchor’ stability to the latent construct, allowing for a reliable estimation of model parameters even with moderate sample sizes.

Another limitation of our study stems from the sample including considerably more women than men. However, in our study, we found no significant differences in Psy-Flex scores between males and females or as a function of age. These findings are consistent with the independence of this construct from non-psychological variables and suggest the divergent validity of the Psy-Flex in line with Gloster et al.'s (2021) hypotheses.

## Conclusion

The Spanish translation of the Psy-Flex by Ruiz et al. (2021) presents a single-factor structure and good psychometric properties of reliability and validity. The scale is also conceptually coherent with the definition of psychological flexibility, as understood in ACT and, more generally, in the field of contextual behavioral science. All this, together with its brevity and context-sensitivity, makes it a tool that contribute to solve the problems of previous instruments and has a great potential for evaluation in research and clinical and health psychology contexts.

## Data Availability

The datasets presented in this article are not readily available because this is an ongoing project. The data that support the findings of this study are available from the corresponding author upon reasonable request. Requests to access the datasets should be directed to Antonio Crego, acregodi@upsa.es.
